# Utilizing the pH-Shift Method for Isolation and Nutritional Characterization of Mantis Shrimp (*Oratosquilla nepa*) Protein: A Strategy for Developing Value-Added Ingredients

**DOI:** 10.3390/foods13152312

**Published:** 2024-07-23

**Authors:** Kanchanaphon Chumthong, Nisa Saelee, Worawan Panpipat, Atikorn Panya, Natthaporn Phonsatta, Sujichon Thangvichien, Wannasa Mala-in, Lutz Grossmann, Manat Chaijan

**Affiliations:** 1 Food Technology and Innovation Research Center of Excellence, School of Agricultural Technology and Food Industry, Walailak University, Nakhon Si Thammarat 80160, Thailand; kanchanaphonchumthong@gmail.com (K.C.); snisa@wu.ac.th (N.S.); pworawan@wu.ac.th (W.P.); 2 Food Biotechnology Research Team, Functional Ingredients and Food Innovation Research Group, National Center for Genetic Engineering and Biotechnology (BIOTEC), National Science and Technology Development Agency, Bangkok 12120, Thailand; atikorn.pan@biotec.or.th (A.P.); natthaporn.pho@biotec.or.th (N.P.); sujichon.tha@ncr.nstda.or.th (S.T.); wannasa.mal@ncr.nstda.or.th (W.M.-i.); 3 Department of Food Science, University of Massachusetts Amherst, 102 Holdsworth Way, Amherst, MA 01003, USA; lkgrossmann@umass.edu

**Keywords:** protein isolate, pH-shift, mantis shrimp, food ingredient

## Abstract

This study focused on the production of protein isolates from mantis shrimp (MS). The pH-shift method was investigated to understand its impact on the protein yield, quality, and properties of the produced isolates. The first step was determining how the pH affected the protein solubility profile, zeta potential, and brown discoloration. The pH-shift process was then established based on the maximum and minimum protein solubilization. The solubilization pH had a significant impact on the mass yield and color of the produced protein, with a pH of 1.0 producing the maximum mass in the acidic region, whereas a maximum was found at a pH of 12.0 in the alkaline region (*p* < 0.05). Both approaches yielded mantis shrimp protein isolates (MPIs) with precipitation at a pH of 4.0 and a mass yield of around 25% (dw). The TCA-soluble peptide and TBARS levels were significantly lower in the MPI samples compared to MS raw material (*p* < 0.05). The MPIs maintained essential amino acid index (EAAI) values greater than 90%, indicating a high protein quality, and the pH-shift procedure had no negative impact on the protein quality, as indicated by comparable EAAI values between the mantis shrimp protein isolate extract acid (MPI-Ac), mantis shrimp protein isolate extract alkaline (MPI-Al), and MS raw material. Overall, the pH-shift approach effectively produced protein isolates with favorable quality and nutritional attributes.

## 1. Introduction

Mantis shrimp (MS; *Oratosquilla nepa*) are crustaceans that are predominantly found in Southern Thailand, and there is a high demand for them both domestically and internationally [1]. In 2021, the annual harvest of MS species in Thailand’s marine waters was 700–800 tons, with a value of 1.18 million USD [2]. The flesh of MS has a mildly sweet flavor and a delicate texture. However, raw MS are susceptible to flesh softening caused by enzymatic and bacterial activities, which reduces their shelf life and market acceptability [3]. This fast spoilage is typically accompanied by the formation of an unpleasant odor and discoloration, which often leads to consumer rejection [4]. Their limited shelf life creates a high pressure on the supply chain and, as a result, increases food waste. Therefore, other food valorization opportunities would expand the possible applications of MS and minimize food waste. 

Chaijan and Panpipat [1] reported the proximate composition of normal and egg-bearing whole MS, including their protein, fat, ash, moisture, and fiber contents. The compositions of both MS types were identical and reported as follows: moisture (78.7–81.3%), protein (11.9–15.2%), fiber (3.5–4.4%), fat (1.3–1.4%), and ash (1.2–1.3%). This demonstrates MS’s potential as a valuable protein source. However, in contrast to regular shrimp (e.g., *Litopenaeus setiferus*), the shells of MS pose a particularly difficult challenge in terms of separation. The covalent bonding of proteins with chitin fibrils directs the conformation and enhances the unique properties of the MS, further complicating the extraction process [5]. Approximately 40% of the MS’s body weight is converted into waste, primarily composed of chitin, lipids, proteins, calcium carbonate, and pigments [6].

The presence of shell and cell-wall material in non-refined ingredients limits the use of whole processed MS as a food ingredient. Therefore, it is important to establish methods that enable the extraction and refinement of the MS proteins to maximize their usability. The successful development of protein extraction methods could not only increase MS consumption but also enhance the overall value of this marine resource. The application of pH-shift protein separation technology appears to be a potential strategy toward achieving this goal and has not been studied before for MS raw materials [7].

This technique is based on solubilization via acid and/or alkaline treatment, followed by isoelectric precipitation, also known as pH-shift processing. Typically, either a high pH (>10.5) or a low pH (<3.5) is used to solubilize muscle proteins, followed by centrifugation to separate the soluble proteins from the insoluble components [8]. The soluble proteins are recovered using isoelectric precipitation and then recovered through centrifugation or filtration [8]. This method presents several benefits, such as achieving a high protein yield and selectively isolating proteins based on the inherent relationship between the protein charge and pH, a relationship that is largely absent in lipids and carbohydrates. The pH-shift method has already been successfully applied to different marine raw materials, including whole fish, cut fish, boned or bone-rich fish, tiny fish, and leftover materials such as fish heads and fishbone trimmings [9]. Studies have demonstrated that, by employing solubilization and precipitation at the isoelectric point, contaminants commonly present in fish muscle, such as dioxins and polychlorinated biphenyls, can be reduced [10]. Moreover, it has been used for mud deodorization in catfish meat and tilapia to eliminate main odorants (geosmin and 2-MIB), as well as protein refinement and salt removal in salted egg white [11,12,13,14,15]. Focus has recently been placed on improving the post-harvest quality of whole MS [2,3] and functional properties of MS myofibrillar proteins [16], but there has not been much research on standardizing the processes for extracting proteins from MS for future application. Therefore, the objective of this study was to isolate and characterize proteins from whole MS using a pH-shifting method to strategically valorize this protein resource.

## 2. Materials and Methods

### 2.1. Chemicals and Raw Materials

The chemicals used in this study were all purchased from Sigma Aldrich Co. (St. Louis, MO, USA).

MS (*O. nepa*) samples, measuring approximately 18–20 cm in total length, were collected from the Thasala coast in the Gulf of Thailand using trawl nets. In order to preserve their freshness, the captured MS were placed on ice within a polystyrene foam container with a MS-to-ice ratio of 1:2 (*w*/*w*) and transported to the laboratory within 30 min. Following a cold tap water rinse, the samples were ground in a bowl chopper at a temperature of 4 °C for a duration of 5 min, resulting in a homogeneous paste. Subsequently, the ground samples were vacuum packed and stored at a temperature of −80 °C for a maximum period of one month prior to their use in experimental procedures. The AOAC [17] method was used to determine the moisture content of ground MS, which was 84%.

### 2.2. pH-Dependent Properties

*Solubility*. Using the approach of Harrysson et al. [18], ground MS (5 g) was mixed with 30 mL of cold distilled water (4 °C) and homogenized for 5 min to facilitate stirring and maximize solubility. High-shear blending was carried out in intervals with 30 s of homogenization followed by 30 s of rest using an IKA high shear dispenser at 20,000 rpm (Model T25 digital Ultra-Turrax, Staufen, Germany). The pH of the homogenate was adjusted to 1–14, using 2 M HCl or 2 M NaOH, as measured by a Cyberscan 500 pH meter (Eutech, Singapore). The mixture was centrifuged at 8500× *g* for 20 min at 4 °C (RC-5B Plus, Sorvall, Norwalk, CT, USA) after 10 min at each pH level. The supernatant was collected and subjected to analysis. The soluble protein level in the supernatant at each pH was determined using the Biuret method [19] and a standard curve was created using bovine serum albumin (BSA) at concentrations ranging from 0 to 10 mg/mL. The protein solubility was reported as g/100 g sample.

*Zeta potential.* The supernatant at each pH was also subjected to zeta potential analysis to monitor the surface charges of the protein solutions, using the Zetasizer Nano-ZS90 (Malvern Instruments Ltd., Worcestershire, UK). The folded capillary cuvette was used.

*Spectrophotometry.* The samples’ turbidity (measured at 660 nm) and brown discoloration (measured at 420 nm) were assessed using a Shimadzu UV-2100 spectrophotometer (Shimadzu Scientific Instruments Inc., Columbia, MD, USA), with deionized water serving as a blank. 

The data obtained above were used to determine the optimal pH for the pH-shift process. In the pH-shift procedure, the pH range with the highest protein solubility and the largest negative or positive zeta potential was defined as the solubilization pH, while the pH with the lowest protein solubility and a zeta potential approaching 0 was termed the precipitation pH.

### 2.3. pH-Shift Protein Isolation 

The mantis shrimp protein isolate (MPI) was obtained using the method of Marmon and Undeland [20]. The aqueous homogenate of ground MS was prepared as detailed above. Based on the solubility profile and zeta-potential results, protein solubilization in the acidic and alkaline pH was investigated. The homogenate’s pH values were adjusted to pH 1.0, 2.0, and 3.0, as well as to pH 10.0, 11.0, and 12.0, using 2 M HCl or 2 M NaOH. The homogenate was stirred continuously at each solubilization pH for 10 min at 100 rpm using a magnetic stirrer. Then, centrifugation (8500× *g*, 4 °C, 20 min) was used to separate the solubilized proteins from the insoluble compounds. The supernatant was subsequently filtered through 3 layers of filter paper. Finally, the pH of the supernatant was adjusted to pH 4 (pI) with 2 M HCl or 2 M NaOH and centrifuged at 8500× *g*, 4 °C, 20 min. The precipitate—known as MPI—was collected, weighed, and analyzed.

### 2.4. Mass Yield

The following formula was used to calculate mass yield [21].

(1) $$\mathrm{Mass}\;\mathrm{yield}\;(\%)=\frac{Weight\;of\;recovered\;protein\;isolate\;\left(g\right)\left(dry\;weight\right)}{Weight\;of\;mince\;(g)\;(dry\;weight)}\times 100$$

### 2.5. Color 

The color of the samples was determined by measuring the L*, a*, and b* values using a Hunterlab Miniscan/EX instrument with 10 standard observers and a standard illuminant D65 (Hunter Assoc. Laboratory, Reston, VA, USA). The redness index (a*/b*) of the sample was determined and the whiteness was calculated using the following formula:

(2) $$\mathrm{Whiteness}=100-\sqrt{\left[{\left(100-L*\right)}^{2}+a{*}^{2}+b{*}^{2}\right]}$$

### 2.6. Determination of Trichloroacetic Acid (TCA)-Soluble Peptides

The content of TCA-soluble peptides was determined using the method of Panpipat and Chaijan [9]. Briefly, 2 g of material was mixed with 16 mL of 5% TCA (*w*/*v*) and homogenized at 11,000 rpm for 2 min. The homogenate was incubated at 4 °C for 1 h and centrifuged at 8000× *g* for 5 min at 25 °C. The TCA-soluble peptides in the supernatant were quantified using the Lowry method [22] and expressed as μmol of tyrosine per g of sample. For calibration, a standard curve was created using tyrosine at concentrations ranging from 0 to 200 μg/mL.

### 2.7. Determination of Thiobarbituric Acid Reactive Substances (TBARS)

The sample (0.5 g) was homogenized with 2.5 mL of a 0.375% (*w*/*v*) thiobarbituric acid, 15% (*w*/*v*) TCA, and 0.25 N HCl solution. The mixture was heated in a boiling water bath (95–100 °C) for 10 min, then cooled with running tap water, and centrifuged at 3600× *g* for 20 min at 25 °C. At 532 nm, the absorbance of the supernatant was read. A standard curve was made using 1,1,3,3-tetramethoxypropane at values ranging from 0 to 10 ppm. TBARS value was expressed as mg of malonaldehyde (MDA) equivalent per kg of sample [23].

### 2.8. Sodium Dodecyl Sulfate-Polyacrylamide Gel Electrophoresis (SDS-PAGE) 

The protein pattern of the MPI samples was examined using SDS-PAGE, using the methodology described by Laemmli [24]. A protein molecular weight standard (pre-stained dual-color standard, 10–250 kDa, Bio-Rad, Hercules, CA, USA) of 10 μL was used, along with 15 μg of protein from each sample. The electrophoresis procedure was conducted using a Mini-Protean II unit (Bio-Rad, USA). After the separation process, the gel was subjected to staining using a solution consisting of 0.02% (*w*/*v*) Coomassie Brilliant Blue R-250 in a mixture of 50% (*v*/*v*) methanol and 7.5% (*v*/*v*) acetic acid. Destaining was carried out by immersing the gel in a solution containing 50% methanol (*v*/*v*) and 7.5% (*v*/*v*) acetic acid for a duration of 1 h. Subsequently, the gel was placed between two layers of cellophane sheets, which were further reinforced by a glass frame. The gel was then subjected to a 24 h period of air drying at ambient temperature (27–30 °C). Following this, the gel image was acquired with a digital camera.

### 2.9. Fourier Transform Infrared (FTIR) Spectra

FTIR analysis was carried out using the method given by Chaijan and Panpipat [11]. The spectra of the dried sample (~0.5 g) were recorded in the range from 400 to 4000 cm^−1^ at a measuring resolution of 4 cm^−1^ over the course of 16 scans. The spectral data were analyzed using the OPUS 3.0 data collection software program (Bruker Co., Ettlingen, Germany). This study used automatic baseline correction techniques in the FTIR analysis to ensure accurate spectrum interpretation.

### 2.10. Amino Acid Composition

Total amino acid and free amino acid compositions were examined using the method given by Chinarak et al. [25]. The amino acid compositions were used to calculate the quantities of essential amino acids (EAA), hydrophobic amino acids, positively charged amino acids, and negatively charged amino acids at pH 7. The essential amino acids index (EAAI) and amino acid score (AAS) were also calculated based on the recommended amino acid profile for adults, according to the Joint WHO/FAO/UNU Expert Consultation [26].

### 2.11. Statistical Analysis

All experiments were performed in triplicate (*n* = 3). Data are provided as mean ± standard deviation. The statistical method utilized was one-way ANOVA. To discover significant differences between the variables, Duncan’s multiple range post hoc test was applied. Differences with probabilities less than 0.05 (*p* < 0.05) were considered statistically significant.

## 3. Results and Discussion

### 3.1. Effect of pH on Protein Solubility, Zeta Potential, Turbidity, and Discoloration

**Figure 1:**
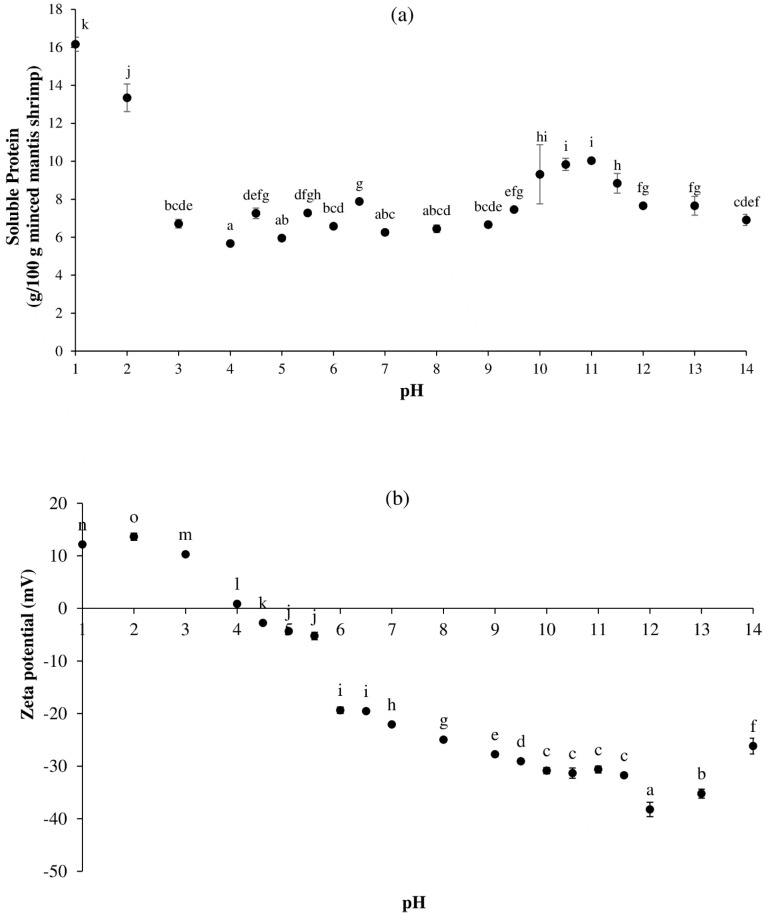
Effect of pH adjustment on protein solubility (**a**) and zeta-potential (**b**) profiles of mantis shrimp (MS) protein. The bars show the standard deviations from triplicate determinations. Different letters indicate the significant differences (*p* < 0.05).

Figure 1a depicts the pH-solubility profile of mantis shrimp protein. The maximum soluble protein of 16.17 g/100 g was discovered at a pH of 1.0 (*p* < 0.05). When the pH of a protein solution was raised from 1.0 to 4.0, the amount of soluble protein gradually declined, eventually reaching its lowest point of 5.67 g/100 g at a pH of 4.0. Beyond a pH of 4.0, the solubility remained almost constant up to a pH of 9.0. This could be due to the fluctuation in the pH solubility profile caused by the presence of various proteins in the MS, as indicated by the SDS-PAGE data (see below) and noted by Chaijan et al. [27]. When the pH was increased higher than 9.0, the solubility steadily increased up to a pH of 11.0 before decreasing until the pH was 14.0.

Overall, a pH of 1.0 had the highest protein solubility in the acidic pH range (*p* < 0.05), whereas pH values of 10.0–11.0 resulted in the highest protein solubility in the alkaline range (*p* < 0.05). The pH-solubility profile of whole mantis shrimp proteins revealed a towards-the-alkaline-region flattened U-shaped solubility profile. U-shape solubility profiles have also been reported for many other species, such as Pacific whiting [28], pig brain [29], and sago palm weevil larvae [27]. However, in certain investigations, the maximal protein solubility in the acidic region was observed at higher pH values of around 3.0 [30,31,32], and, in the alkaline region, at higher values of around a pH of 12.0 [33,34,35]. This suggests that the pH solubility profile of muscle proteins is species-dependent. Nevertheless, such outcomes could also be influenced by varying processing conditions, such as the centrifugation speed, or by employing different protein analysis techniques.

From the solubility measurements, no clear isoelectric point could be identified. To reveal the pI of mantis shrimp protein, the zeta potentials of protein solutions at different pH values were measured (Figure 1b). Zeta potentials are commonly used to describe the surface potential properties of proteins as well as their molecular interactions [36]. The higher the absolute value of the zeta potential, the stronger the electrostatic repulsion on the protein surface is between similarly charged particles, which results in a more stable protein system and a lower aggregation probability [36].

Positively charged proteins were discovered at pH values ranging from 1.0 to 4.0, with a pH of 2.0 having the highest charge (*p* < 0.05). At pH values between 4.5 and 14.0, proteins had negative charges, with a pH of 12.0 having the highest negative charge (*p* < 0.05). At a pH of 4.0, the zeta potential was close to 0, indicating that the majority of mantis shrimp proteins had a pI around this pH value. It has been reported that the pI of white shrimp (*Litopenaeus vannamei*) muscle protein was a pH of 4 [37], while Cremades et al. [38] discovered that the pI of crayfish (*Procambarus clarkii*) muscle protein was at around 4.3.

In the acidic pH range, a pH of 2.0 possessed the highest positive zeta potential (*p* < 0.05), followed by a pH of 1.0. In the alkaline range, a pH of 12.0 had the maximum negative zeta potential (*p* < 0.05), while a decrease was observed when the pH was further increased to 13.0–14.0. This is most likely related to the strong intramolecular repulsive forces at very alkaline conditions, which can result in protein denaturation. The unfolding can alter the surface composition, consequently impacting the zeta potential and leading to a decrease in protein solubility (Figure 1a) [29,36].

**Figure 2:**
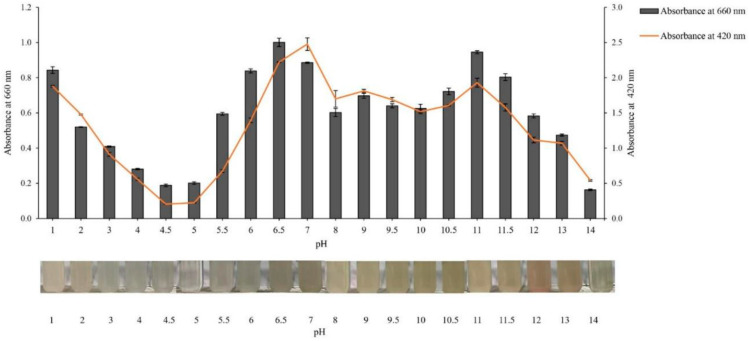
Effect of pH adjustment on turbidity (absorbance at 660 nm) and browning intensity (absorbance at 420 nm) profiles of mantis shrimp (MS) protein. The bars show the standard deviations from triplicate determinations.

The degree of protein aggregation is often analyzed using turbidity, which can be measured as the absorbance at 660 nm [39]. Due to the possibility of both enzymatic and non-enzymatic browning reactions at various pHs, absorbance at 420 nm was employed as a measure of the browning intensity [40]. The results (Figure 2) showed a similar pattern for the turbidity and browning intensity. These patterns also resembled the pH-solubility profile (Figure 1a). It is possible that the amount of protein in protein solutions at various pH values is related to the turbidity and browning intensity. Based on the results obtained from the solubility, zeta potential, browning intensity, and turbidity measurements, as well as previous information from other studies, pH values of 1.0, 2.0, and 3.0 (acid solubilization) and pH values of 10.0, 11.0, and 12.0 (alkaline solubilization) were used to extract protein from MS, while a pH of 4.0 was used for precipitation in the following steps.

### 3.2. Characteristics of Mantis Shrimp Protein Isolates (MPI) Prepared at Different Solubilization pH

#### 3.2.1. Mass Yield and Color

The mass yield or recovery yield is an essential factor in determining how much protein or mass can be recovered in an extraction process, which determines the economic viability. The solubilization pH had a significant impact on the mass yield (Table 1). Employing acidic solubilization, a pH of 1.0 resulted in the highest mass yield (*p* < 0.05), whereas a pH of 12.0 resulted in the highest mass yield when alkaline solubilization was used (*p* < 0.05) (Table 1). Both procedures produced around a 25% mass yield (dw). In research on another crustacean, Abreu et al. [37] obtained a 9.8% (dw) protein yield using the pH-shifting (solubilization at pH 7 and precipitation at pH 4) of fresh cephalothorax and exoskeleton byproducts of white shrimp (*L. vannamei*).

In terms of color, the MPI solubilized at a pH of 1.0 had the highest whiteness value, while solubilization at a pH of 12.0 generated the lowest whiteness. Furthermore, proteins extracted at a pH of 12.0 displayed the highest redness index compared to other solubilization pH values (*p* < 0.05). One possible explanation for this phenomenon is the solubilization of carotenoprotein at extreme alkaline pH levels. Furthermore, it has been shown that an exceptionally high pH level might cause the denaturation of carotenoproteins, resulting in the exposure of unbound carotenoids (notably astaxanthin) and, consequently, in a red appearance [41].

#### 3.2.2. TCA-Soluble Peptide

**Figure 3:**
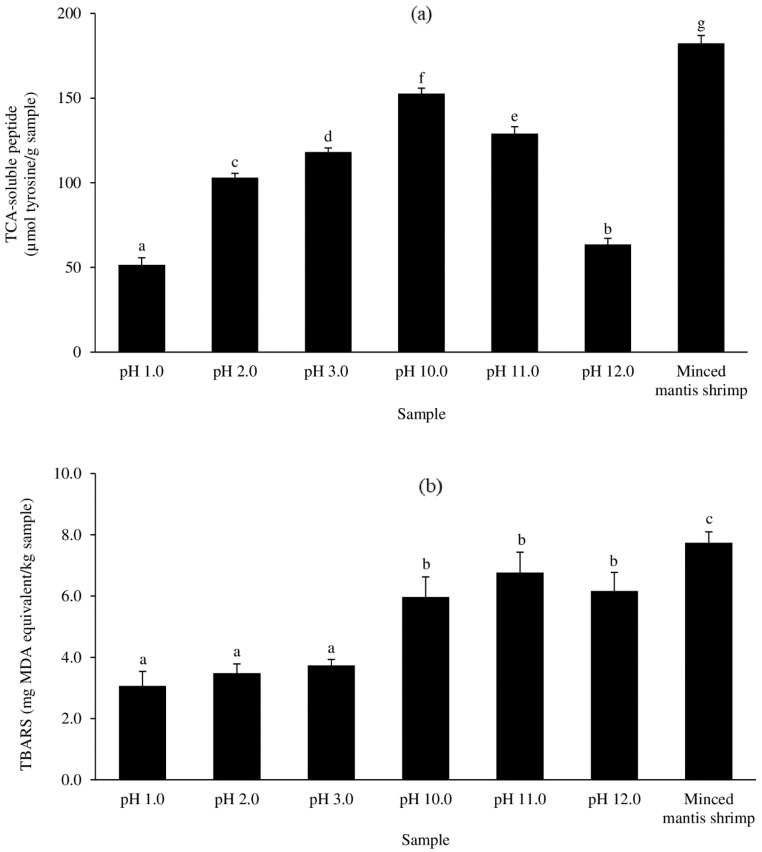
TCA-soluble peptide (**a**) and TBARS (**b**) values of acid-aided (pH 1.0, 2.0, and 3.0), alkaline-aided (pH 10.0, 11.0, and 12.0), and MS raw materials. Bars represent the standard deviations from triplicate determinations. Different letters indicate the significant differences (*p* < 0.05).

The TCA-soluble peptide levels in the MPI samples were much lower than in the MS raw material (*p* < 0.05) (Figure 3a). The lowest TCA-soluble peptide values were identified in MPIs produced by solubilization at a pH of 1.0 (*p* < 0.05), followed by solubilization at a pH of 12.0 (Figure 3a). The lower the pH that was used for the acid-solubilization to obtain MPIs, the lower the TCA-soluble peptide content was. Likewise, the higher the pH during alkaline solubilization for MPI production, the lower the TCA-soluble peptide content was. Both methods of solubilization (acidic and alkaline) may have partially inactivated proteases during the pH adjustment, resulting in fewer TCA-soluble peptides remaining in both isolates [9,42]. It has been found that raising the pH from 10 to 12 considerably reduces the relative activity of alkaline proteases from goby (*Zosterisessor ophiocephalus*), thornback ray (*Raja clavata*), and scorpionfish (*Scorpaena scrofa*) [43].

#### 3.2.3. TBARS 

The MS raw material had the highest TBARS level, followed by the MPIs produced through alkaline solubilization and acid solubilization, but no difference was found within the two regimes (*p* > 0.05) (Figure 3b). As a result, the pH-shift processing reduced the presence of lipid oxidation end products when compared to the MS raw material. This result could be related to the use of the pH adjustment method, which facilitates the removal of fat and prooxidant components from the sample through solubilization and centrifugation [9]. Moreover, the carotenoproteins that were solubilized in the alkaline regime (see Section 3.2.1) and which were found in the respective MPIs may have undergone some additional oxidation during the processing, and the carotenoid-oxidized products might have exacerbated the lipid oxidation. Although astaxanthin is a natural antioxidant, due to its unsaturated nature, it can also be oxidized. Astaxanthin degradation and lipid oxidation in oil from the hepatopancreas of Pacific white shrimp (*Litopenaeus vannamei*) happened simultaneously during storage, as reported by Takeungwongtrakul and Benjakul [44]. Thus, the degradation of astaxanthin and lipid oxidation could further contribute to the formation of MDA and other oxidation products.

Based on the yields and because there was no significant difference in the TBARS values within the two solubilization regimes, a pH of 1.0 was chosen for the mantis shrimp protein isolate extract acid (MPI-Ac) process and a pH of 12.0 for the mantis shrimp protein isolate extract alkaline (MPI-Al) process during the solubilization step.

### 3.3. Molecular Properties and Amino Acid Composition of MPIs

#### 3.3.1. SDS-PAGE 

**Figure 4:**
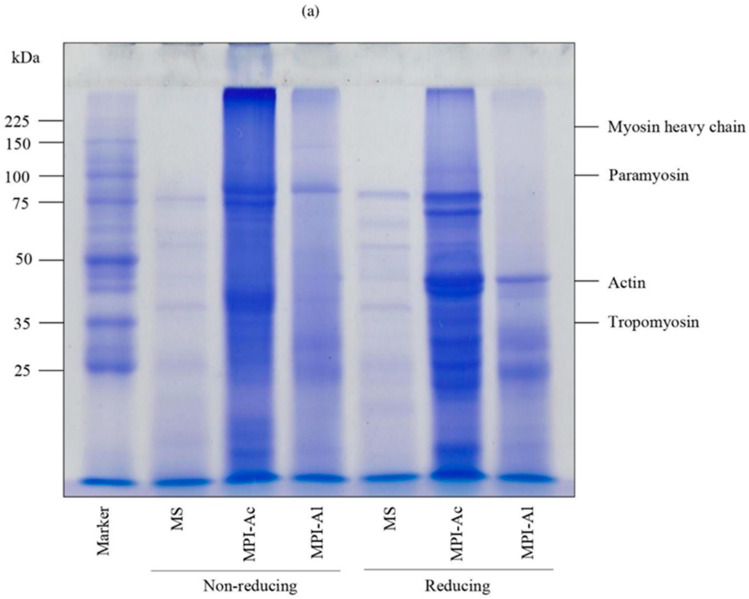

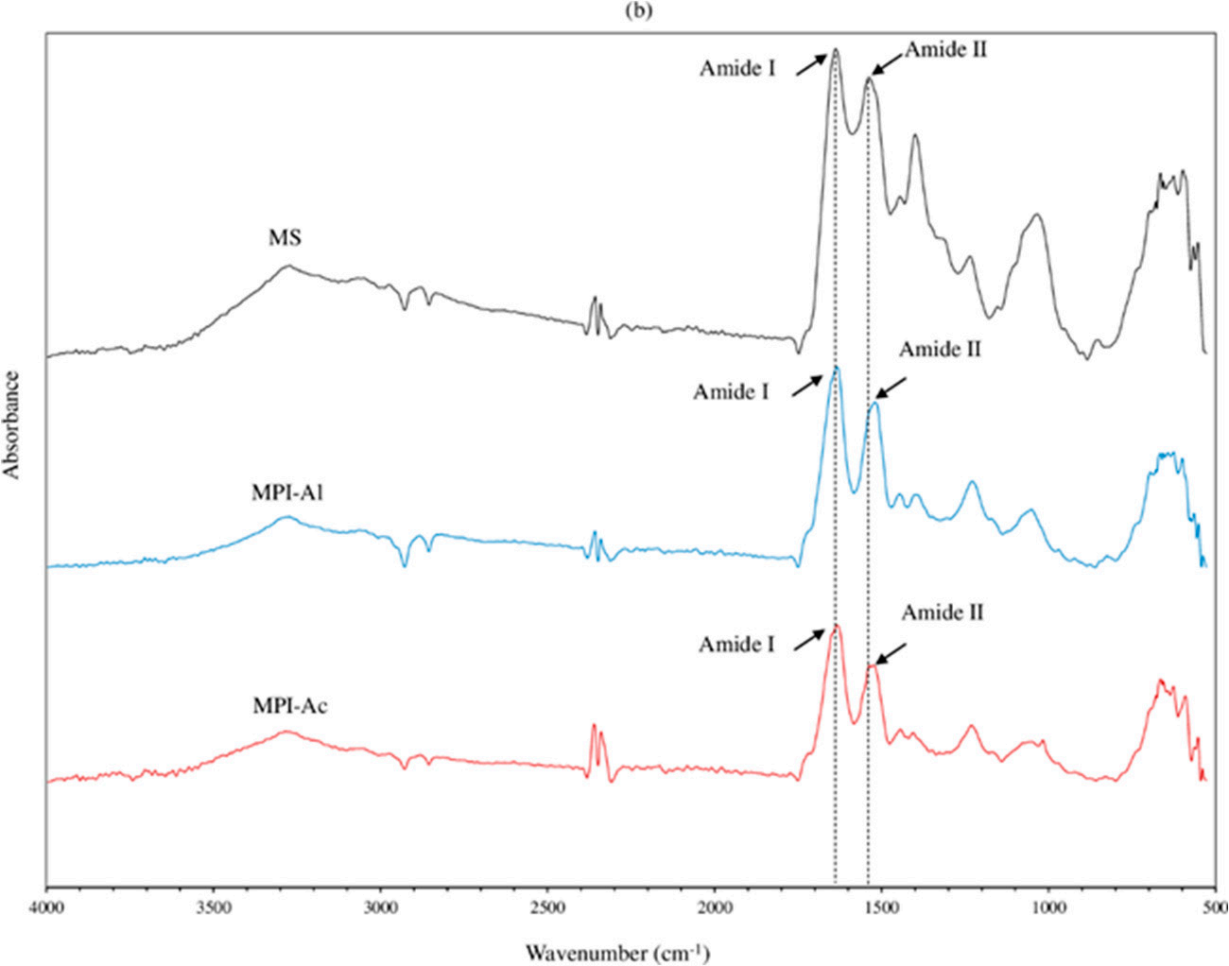
Sodium dodesylsulfate-polyacrylamide gel electrophoresis (SDS-PAGE) patterns of MS (proteins from minced mantis shrimp) (Lane 1), MPI-Ac (mantis shrimp protein isolate extract acid) (Lane 2), and MPI-Al (mantis shrimp protein isolate extract alkaline) (Lane 3) under non-reducing (without β-ME) and reducing (with β-ME) conditions (Marker = protein markers) (**a**) and Fourier transform infrared (FTIR) spectra of the freeze-dried MPI-Ac, the freeze-dried MPI-Al and, the freeze-dried minced MS material (**b**).

Figure 4a shows the SDS-PAGE protein patterns of MPIs under reducing and non-reducing conditions as well as those of the untreated MS. In general, the bands for myosin heavy chain (MHC), paramyosin, actin, and tropomyosin have molecular weights of about 200, 100, 45, and 35 kDa, respectively [45]. The results showed that the protein patterns in all samples were relatively comparable, albeit with variations in their band intensities.

Lane 1 in Figure 4a shows a protein pattern in the MS raw material, with prominent bands at molecular weights of 75, 55, 45, 37, and 25 kDa or smaller. Muscle proteins can be divided into three fractions: myofibrillar proteins, sarcoplasmic proteins, and stromal proteins [27]. The MHC band, which normally has a molecular weight of about 200 kDa, was absent in the MS raw material. Given that the endogenous proteases may cause the MHC to hydrolyze, the observed effect may be explained by protein degradation [46]. Thus, it is possible that proteolysis is responsible for the low molecular weight bands that were observed. Chanchi Prashanthkumar et al. [47] reported that harpiosquillid MS (*Harpiosquilla raphidea*) quality degradation and customer rejection are largely related to the quick texture softening induced by proteolysis after harvesting. This could explain why the molecular weights of most proteins discovered in MS are comparatively low.

More bands were identified in the protein extracts, which could be related to lower protein degradation compared to the untreated MS. Bands were identified in the MPI-Ac (Figure 4a, Lane 2) with molecular weights of 25, 37, 45, and 70–80 kDa, as well as the bands of high MW proteins with >225 kDa that did not migrate into gel. These high MW proteins might have been produced during the protein extraction through protein denaturation. The alkali-aided MPI (Figure 4a, Lane 3) exhibited the existence of major bands with molecular weights at 25, 37, 45, 80, and 150 kDa, and a small fraction of proteins larger than 225 kDa. The myosin light chain may be represented by the band at 25 kDa. Actin and tropomyosin could be the bands with molecular weights of 45 and 37 kDa, respectively [45]. Moreover, hemocyanin, which is a blue respiratory protein with an estimated molecular weight of 73.1–75.1 kDa, might be present in MPI [48]. 

There was a discernible difference in the protein patterns exhibited between the non-reducing and reducing conditions of the MPI-Ac and MPI-Al procedures. The existence of disulfide bonds (which are cleaved under reducing conditions) in the protein structures and aggregates in the MPIs obtained through both pH-shift procedures may contribute to the different protein patterns observed. Disulfide bonds have been suggested to be involved in the aggregate formation that may occur during pH-shift procedures in a range of raw materials, both in alkaline- and acid-assisted conditions [27].

#### 3.3.2. FTIR Spectroscopy

Figure 4b shows the FTIR spectra of the MPI-Ac, the MPI-Al, and the MS raw material. Notably, the characteristic peaks at 1600–1700 cm^−1^ (corresponding to C = O stretching) and 1480–1575 cm^−1^ (indicative of CN stretching and NH bending) are identified as amide I and amide II bands [49]. Basically, the amide I band can be related to the secondary structure profile of proteins. The wavenumbers associated with different secondary structures in proteins are as follows: β-sheet (1610–1640 and 1670–1690 cm^−1^), α-helix (1650–1660 cm^−1^), random coil (1640–1650 cm^−1^), and β-turn (1660–1670 and 1690–1700 cm^−1^) [21,50]. In this study, amide I and II bands were consistently observed in all samples, falling within the wavenumber ranges of 1633–1637 cm^−1^ and 1519–1537 cm^−1^, respectively. Specifically, the MPI-Ac exhibited an amide I band at a wavenumber of 1633 cm^−1^ and an amide II band at 1523 cm^−1^. Conversely, the MPI-Al displayed an amide I band at 1633 cm^−1^ and an amide II band at 1519 cm^−1^. In contrast, the MS raw material showcased an amide I band at a wavenumber of 1637 cm^−1^ and an amide II band at 1537 cm^−1^. These wavenumber shifts between the MS raw material and the MPI-Ac and MPI-Al can be attributed to potential secondary structural changes induced by the pH-shift process. This shift is likely due to the unfolding of the protein structure under extreme pH conditions, as reported by Zhao et al. [51]. Furthermore, in FTIR spectroscopy, the peak intensity represents the maximum absorption of infrared radiation by a sample at a specific wavenumber. The analysis revealed that the peak intensities of the amide I and II bands in the MPI-Al and MPI-Ac were lower than that of the MS raw mantis shrimp. These results suggest that the extraction process may have induced alterations in the protein structures and conformations of the MPI-Al and MPI-Ac samples. Such changes can impact the secondary structure and conformation of the proteins, leading to a decrease in peak intensity. Additionally, the peak intensity of MPI-Ac was lower than that of MPI-Al, indicating a possible difference in the secondary structure composition between the acid and alkaline extraction processes. This observation highlights the importance of a solubilization pH during the extraction process, as variations in the pH can affect the protein solubility and structure, ultimately impacting the functional properties of the isolated proteins. To gain a comprehensive understanding of these changes and their implications for the physicochemical and functional properties of the MPIs, more research into their functional properties is needed. Additionally, noteworthy absorption peaks were identified in the MS raw material spectra, particularly at 1400 and 855 cm^−1^, corresponding to CO_3_^2−^ stretching and bending vibrations, indicating the presence of CaCO_3_ (calcite). Furthermore, a peak at 1035 cm^−1^, indicative of a C-O-C glycosidic bond, offers insight into the components of the shell, including calcite and chitin [6,52].

#### 3.3.3. Amino Acid Composition

The amino acid profiles of the ingredients influence both their nutritional value and functional properties [53]. Table 2 shows the total and free amino acid compositions of MPI-Ac, MPI-Al, and MS raw material.

In terms of total amino acid composition, glutamic acid was the major amino acid in both the MPI-Ac and MPI-Al, while tryptophan was the predominant amino acid and the most abundant essential amino acid (EAA) in the MS raw material. Furthermore, both MPIs contained a high concentration of glutamic acid and aspartic acid, which was associated with a high percentage of negatively charged amino acids, assuming a pH of 7. This finding was similar to the results of the study conducted by Zhang et al. [54]. In addition, the MPI-Ac had a lower total amino acid content than the MPI-Al. This indicates that the true protein content in the MPI-Ac is lower, possibly due to the co-extraction of other components under acidic conditions [55]. 

Interestingly, MPI contains non-proteinogenic amino acids like ornithine. Studies have indicated that ornithine has bioactive properties such as sedative and hypnotic effects in rats subjected to acute stress [56,57] and fatigue reduction by enhancing energy efficiency and promoting ammonia excretion [58]. In general, free amino acids made up 0.67%, 0.60%, and 7.36% of the total amino acid content in the MPI-Ac, MPI-Al, and MS raw materials, respectively. Such free amino acids may enhance the taste of food by adding umami taste notes, for example [59]. The MPI-Ac and MPI-Al showed reduced quantities of free amino acids compared to the raw material (*p* < 0.05), consistent with Admassu et al. [60]. The results aligned with the TCA-soluble peptide content (Figure 3a), indicating that pH-shift processing can prevent proteolysis and thus lower the content of free amino acids in the final MPI. 

The content and location of hydrophilic and hydrophobic amino acids have a major impact on the functionality of proteins, particularly their surface composition [27]. A protein’s hydrophilic to hydrophobic balance is directly related to its efficiency as an emulsifier [61]. The MPI-Ac and MPI-Al contained approximately 45% hydrophobic amino acids, whereas higher values were found in the MS raw material, with up to 50% hydrophobic amino acids (Table 2). This indicates that more hydrophilic proteins were solubilized during the extraction process. Moreover, the amino acid composition of the raw MS protein, particularly hydrophilic amino acids like glutamic and aspartic acid, has a substantial influence on its solubility profile [27], with these amino acids being the most abundant in MPIs (Table 2).

All of the samples contained all nine EAAs, and the ratio of EAAs to total amino acids was around 43–50% (Table 2), above the Joint WHO/FAO/UNU Expert Consultation (2007)‘s recommended value of 29%. The findings revealed that MS raw material and MPIs contain more EAAs than plant-based protein sources such as seaweed, lupine, faba bean, hemp, and flaxseed [59,62] or insect-based protein, such as protein isolate from sago palm weevil larvae [27]. All samples had comparable quantities of EAAs to casein and pig brain [59,61]. 

The EAAI measures protein quality by comparing the proportion of EAAs in the target protein to that of a reference protein [63]. The EAAIs of MPI-Ac, MPI-Al, and MS raw materials are shown in Table 2. There was no significant difference in the EAAIs between the MPI-Ac, MPI-Al, and MS raw material (*p* < 0.05), indicating that the pH-shift method did not negatively impact the protein quality. A protein is considered to be of high quality when its EAAI value exceeds 90%. It is classified as moderate quality when the EAAI falls between the range of 70 and 89%, and as low quality when the EAAI is below 70% [59]. All samples had EAAI values higher than 90%, therefore indicating high quality.

Furthermore, Table 3 displays the amino acid score (AAS) values for MPI-Ac and MPI-Al compared to the MS raw material based on the Joint WHO/FAO/UNU Expert Consultation’s [26] recommended amino acid pattern for adults. The total EAA values were not significantly different between the MPI-Ac, MPI-Al, and MS raw material (*p* < 0.05). Thus, it is confirmed that the pH-shift process has no negative impact on the nutritional value or quality of protein.

## 4. Conclusions

The study demonstrated that the pH-shift method effectively produced high-quality protein isolates with desirable characteristics and nutritional value. The research findings highlighted that the pH-shift process did not compromise the protein quality, as indicated by high EAAI values exceeding 90% in all samples Acid-aided and alkaline processing showed similar mass yields, suggesting both methods are effective for producing quality protein isolates. The solubilization pH significantly influenced the mass yield, color attributes, lipid oxidation levels, protein structure, and amino acid composition in the protein isolates. The results underscored the importance of pH control in optimizing the protein solubility and quality during processing. Overall, this study provides valuable insights into the efficacy of the pH-shift method in producing protein isolates with favorable properties for further applications. This could pave the way for future studies into the functional properties of these isolated proteins in order to add value and employ them as an alternative protein source.

## Figures and Tables

**Table 1:** Yield and color of protein isolates from whole mantis shrimp produced by the pH-shift method at varying solubilization pH.

Characteristics	Solubilization pH					
	pH 1.0	pH 2.0	pH 3.0	pH 10.0	pH 11.0	pH 12.0
Mass yield (%)	24.89 ± 1.87 ^c^	19.53 ± 3.41 ^b^	5.15 ± 1.80 ^a^	19.91 ± 1.06 ^b^	20.83 ± 1.03 ^b^	25.21 ± 1.52 ^c^
Whiteness	35.90 ± 0.03 ^f^	34.39 ± 0.11 ^d^	35.37 ± 0.06 ^e^	32.36 ± 0.10 ^c^	31.46 ± 0.23 ^b^	26.66 ± 0.13 ^a^
Redness index	0.96 ± 0.03 ^a^	1.04 ± 0.03 ^a^	0.88 ± 0.02 ^a^	0.92 ± 0.07 ^a^	1.13 ± 0.04 ^a^	9.24 ± 1.36 ^b^
Appearance	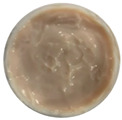	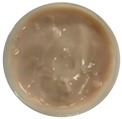	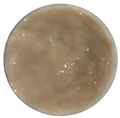	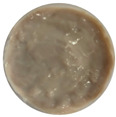	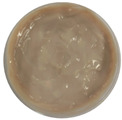	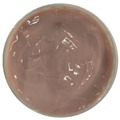

Values are shown as mean ± standard deviation of triplicate determinations. Significant differences are observed between letters in the same row (*p* < 0.05).

**Table 2:** Total amino acid and free amino acid compositions expressed in mg/g of dry sample of minced mantis shrimp, acid-aided protein isolate, and alkaline-aided protein isolate.

Amino Acid (mg/g Sample)	Total Amino Acid			Free Amino Acid		
	Raw Material	Acid-Aided Process	Alkaline-Aided Process	Raw Material	Acid-Aided Process	Alkaline-Aided Process
Essential amino acids (EAA)						
Valine (Val) ^1^	13.91 ± 1.33 ^a^	25.83 ± 0.67 ^b^	27.82 ± 0.71 ^c^	0.40 ± 0.02 ^c^	0.09 ± 0.01 ^b^	0.06 ± 0.00 ^a^
Leucine (Leu) ^1^	20.95 ± 2.06 ^a^	36.06 ± 0.29 ^b^	42.02 ± 0.96 ^c^	1.08 ± 0.18 ^b^	0.18 ± 0.01 ^a^	0.14 ± 0.00 ^a^
Isoleucine (Ile) ^1^	13.98 ± 1.84 ^a^	29.48 ± 0.60 ^b^	32.32 ± 0.68 ^c^	0.52 ± 0.02 ^b^	0.08 ± 0.00 ^a^	0.07 ± 0.00 ^a^
Phenylalanine (Phe) ^1^	11.66 ± 1.14 ^a^	27.40 ± 0.47 ^b^	32.13 ± 1.54 ^c^	1.29 ± 0.58 ^b^	0.16 ± 0.03 ^a^	0.15 ± 0.02 ^a^
Methionine (Met) ^1^	6.38 ± 1.07 ^a^	13.43 ± 0.63 ^b^	15.46 ± 0.97 ^c^	0.44 ± 0.08 ^b^	0.07 ± 0.01 ^a^	0.06 ± 0.01 ^a^
Lysine (Lys) ^2^	30.71 ± 2.10 ^a^	45.85 ± 3.39 ^b^	45.29 ± 3.05 ^b^	3.20 ± 0.39 ^b^	0.38 ± 0.07 ^a^	0.71 ± 0.11 ^a^
Histidine (His) ^2^	20.78 ± 0.65 ^a^	25.84 ± 0.26 ^b^	25.94 ± 0.47 ^b^	0.24 ± 0.01 ^c^	0.08 ± 0.00 ^a^	0.11 ± 0.00 ^b^
Tryptophan (Trp) ^1^	53.05 ± 1.05 ^a^	60.62 ± 5.80 ^a^	59.40 ± 8.55 ^a^	0.91 ± 0.12 ^b^	0.25 ± 0.01 ^a^	0.24 ± 0.00 ^a^
Threonine (Thr)	11.71 ± 0.84 ^a^	26.01 ± 5.67 ^b^	28.24 ± 4.62 ^b^	0.54 ± 0.12 ^b^	0.06 ± 0.01 ^a^	0.07 ± 0.01 ^a^
Non-essential amino acids						
Alanine (Ala) ^1^	21.24 ± 1.85 ^a^	30.34 ± 1.29 ^b^	32.70 ± 1.35 ^b^	1.54 ± 0.06 ^c^	0.46 ± 0.11 ^b^	0.25 ± 0.02 ^a^
Glycine (Gly) ^1^	27.56 ± 1.73 ^a^	41.92 ± 1.48 ^b^	46.01 ± 2.86 ^b^	2.50 ± 0.05 ^c^	0.88 ± 0.09 ^b^	0.41 ± 0.01 ^a^
Proline (Pro) ^1^	15.57 ± 1.46 ^a^	30.79 ± 2.21 ^b^	28.38 ± 5.79 ^b^	0.74 ± 0.02 ^c^	0.16 ± 0.02 ^b^	0.12 ± 0.00 ^a^
Serine (Ser)	8.96 ± 1.35 ^a^	20.42 ± 1.52 ^b^	22.66 ± 0.64 ^b^	0.34 ± 0.03 ^b^	0.07 ± 0.02 ^a^	0.07 ± 0.01 ^a^
Aspartic acid (Asp) ^3^	22.25 ± 4.85 ^a^	79.76 ± 0.45 ^b^	85.99 ± 9.50 ^b^	0.26 ± 0.10 ^b^	0.03 ± 0.00 ^a^	0.06 ± 0.01 ^a^
Cystine (Cys)	5.91 ± 0.97 ^a^	10.78 ± 1.93 ^a^	11.11 ± 4.76 ^a^	0.33 ± 0.02 ^b^	0.28 ± 0.00 ^a^	0.31 ± 0.01 ^b^
Glutamic acid (Glu) ^3^	36.69 ± 5.12 ^a^	87.45 ± 1.68 ^b^	95.71 ± 8.96 ^b^	0.56 ± 0.24 ^b^	0.06 ± 0.02 ^a^	0.12 ± 0.00 ^a^
Glutamine (Gln)	ND	ND	ND	5.70 ± 1.55 ^b^	0.19 ± 0.04 ^a^	0.40 ± 0.02 ^a^
Tyrosine (Tyr)	18.17 ± 1.24 ^a^	44.25 ± 0.47 ^b^	51.12 ± 0.33 ^c^	1.88 ± 0.32 ^b^	0.27 ± 0.06 ^a^	0.24 ± 0.04 ^a^
Arginine (Arg) ^2^	ND	ND	ND	ND	ND	ND
Ornithine (Orn)	21.04 ± 0.89 ^a^	23.83 ± 0.54 ^b^	23.94 ± 0.52 ^b^	2.91 ± 0.63 ^b^	0.30 ± 0.02 ^a^	0.47 ± 0.03 ^a^
Gamma-aminobutyric acid (GABA)	ND	ND	ND	0.02 ± 0.00 ^a^	0.02 ± 0.00 ^a^	ND
Sarcosine (Sar)	3.18 ± 0.38 ^a^	ND	ND	1.34 ± 0.16 ^b^	0.40 ± 0.03 ^a^	0.21 ± 0.02 ^a^
Total amino acids	363.67 ± 31.94 ^a^	660.05 ± 18.84 ^b^	706.23 ± 7.38 ^c^	26.75 ± 2.05 ^b^	4.45 ± 0.20 ^a^	4.26 ± 0.14 ^a^
Hydrophobic amino acids (% of total amino acid)	50.71 ± 0.73 ^b^	44.83 ± 0.64 ^a^	44.77 ± 1.53 ^a^	- *	-	-
Positively charged amino acids (% of total amino acid)	14.18 ± 0.49 ^b^	10.86 ± 0.33 ^a^	10.09 ± 0.53 ^a^	-	-	-
Negatively charged amino acids (% of total amino acid)	16.15 ± 1.33 ^a^	25.35 ± 0.75 ^b^	25.75 ± 2.66 ^b^	-	-	-
EAA (% of total amino acid)	50.40 ± 1.10 ^b^	44.00 ± 0.71 ^a^	43.68 ± 1.99 ^a^	-	-	-
EAAI (%) ^4^	111.43 ± 9.45 ^a^	107.01 ± 5.01 ^a^	107.06 ± 6.98 ^a^	-	-	-

Values are given as mean ± standard deviation from triplicate determinations. Different letters in the same row indicate significant differences between the raw material, acid-aided process, and alkaline-aided process (*p* < 0.05). ND = not detected. ^1^ Hydrophobic amino acid. ^2^ Positively charged amino acids. ^3^ Negatively charged amino acid. ^4^ Cysteine and tyrosine were included in the calculation and the amino acid scoring pattern of a reference protein was obtained from Joint WHO/FAO/UNU Expert Consultation [26]. * These values were not determined for free amino acid composition.

**Table 3:** Amino acid scores (AASs) of minced mantis shrimp raw material, acid-aided protein isolate, and alkaline-aided protein isolate.

Amino Acid (mg Amino Acid/g Protein)	Raw Material	Acid-Aided Process	Alkaline-Aided Process	Reference Protein *
Valine	38.24 ± 3.65 ^a^	39.13 ± 1.02 ^a^	39.39 ± 1.01 ^a^	40.00
	(0.96)	(0.98)	(0.98)	
Leucine	57.61 ± 5.67 ^a^	54.64 ± 0.44 ^a^	59.50 ± 1.36 ^a^	61.00
	(0.94)	(0.90)	(0.98)	
Isoleucine	38.43 ± 5.07 ^a^	44.66 ± 0.91 ^b^	45.76 ± 0.97 ^b^	30.00
	(1.28)	(1.49)	(1.53)	
Threonine	32.21 ± 2.30 ^a^	39.41 ± 8.60 ^a^	39.98 ± 6.53 ^a^	25.00
	(1.29)	(1.58)	(1.60)	
Lysine	84.43 ± 5.79 ^b^	69.46 ± 5.13 ^a^	64.13 ± 4.32 ^a^	48.00
	(1.76)	(1.45)	(1.34)	
Histidine	57.13 ± 1.77 ^c^	39.15 ± 0.39 ^b^	36.73 ± 0.66 ^a^	16.00
	(3.57)	(2.45)	(2.30)	
Tryptophan	145.87 ± 2.90 ^b^	91.83 ± 8.79 ^a^	84.11 ± 12.11 ^a^	6.60
	(22.10)	(13.91)	(12.74)	
Phenylalanine + Tyrosine	82.03 ± 6.56 ^a^	108.55 ± 0.46 ^b^	117.89 ± 2.08 ^c^	41.00
	(2.00)	(2.65)	(2.88)	
Methionine + Cysteine	33.80 ± 5.62 ^a^	36.67 ± 3.43 ^a^	37.62 ± 8.11 ^a^	23.00
	(1.47)	(1.59)	(1.64)	
Total EAA	569.74 ± 39.32 ^a^	523.51 ± 22.85 ^a^	525.10 ± 31.38 ^a^	290.60
	(1.96)	(1.80)	(1.81)	

The amino acid content of the sample was converted to the amino acid content of the total amino acids in the sample and then calculated. Values are given as mean ± standard deviation from triplicate determinations. Different letters in the same row indicate significant differences between the raw material, acid-aided process, and alkaline-aided process (*p* < 0.05). * Joint WHO/FAO/UNU Expert Consultation (2007) [26].

## Data Availability

The original contributions presented in the study are included in the article, further inquiries can be directed to the corresponding author.
